# When Labiaplasty is Not Enough: Rejuvenation of the Labia Majora Using Hybrid Calcium Hydroxylapatite With Hyaluronic Acid Filler for Increased Satisfaction and Uniform Results

**DOI:** 10.1093/asjof/ojag101

**Published:** 2026-06-02

**Authors:** Genevieve F Caron

## Abstract

Dermal fillers hyaluronic acid (HA) blended with calcium hydroxylapatite–carboxymethylcellulose (CaHA–CMC) for facial indications is increasingly being adapted for body applications, offering both volumizing and regenerative benefits to the skin. In its hyperdilute form, CaHA functions as a regenerative biostimulator, effectively inducing the synthesis of key extracellular matrix components, whereas HA provides immediate volumization. Vaginal rejuvenation, with or without surgery, is also gaining attention with reports of off-label use of fillers for labia majora augmentation; however, analyses of hybrid approaches remain limited. This report outlines a technique utilized in 2 patients in which, following labia minora reduction, the labia majora were volumized using a combination of fillers with the aim of improving both skin quality and enhanced volumetric support. Patients who had previously undergone labiaplasty 6 months prior, presenting with mild-to-moderate laxity of the labia majora, received a single treatment of combined CaHA–CMC and HA injected into the subdermal plane. Outcomes were evaluated after 3 and 20 months for skin texture, firmness, adverse effects, and patient satisfaction levels. Posttreatment, improvements to skin quality, flaccidity, volumization, and dermal integrity were observed for both patients. High patient satisfaction was similarly reported, along with no adverse effects nor prolonged downtime typically associated with more invasive surgical alternatives, such as fat grafting. The treatment presented here offers a promising solution for postlabiaplasty patients seeking further labia majora rejuvenation, offering preliminary evidence of the safety and efficacy of hybrid fillers for addressing laxity.

Level of Evidence: 4 (Therapeutic)

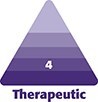

Increasing awareness of sexual health as a critical factor of holistic well-being has emerged as a significant growth driver of the sexual wellness market.^1^ Globally, the sexual wellness market was worth $31.5 billion USD in 2023 and is projected to grow to $58.6 billion USD in 2032 at a compound annual growth rate (CAGR) of 7.15%.^1^ Surgical procedures to enhance the aesthetics of female genitalia, including labiaplasty, clitoral hood reduction, and vaginal fat injections, accounted for nearly 20,000 procedures in 2022.^2^ However, as with all surgical interventions, these procedures carry inherent risks, added cost, and longer postoperative downtime. Similar to trends seen across the broader aesthetics market, consumers are increasingly seeking sexual wellness services that are effective, safe, and require minimal recovery time.

Comparable to the aging process observed in the face, aging of the labia majora is associated with volume loss and increased skin laxity. A few publications have described the use of injectable fillers, including hyaluronic acid (HA) or calcium hydroxylapatite–carboxymethylcellulose (CaHA–CMC) to augment the labia majora and/or mon pubis for aesthetic improvement with high patient satisfaction and little downtime.^3-7^ Calcium hydroxylapatite–carboxymethylcellulose (Radiesse, Merz Aesthetics, Raleigh, NC) is the only semipermanent injectable product that provides both immediate volume and regenerates proteins in the skin resulting in enhanced skin quality and improvements in skin laxity and wrinkles.^8^ Calcium hydroxylapatite–carboxymethylcellulose is comprised of homogeneous calcium hydroxylapatite microspheres suspended in a carboxymethylcellulose (CMC) gel. The CMC gel is metabolized within ∼2 months, which may result in a decrease in the volumizing aspect before newly developed collagens types 1 and 3, elastin, proteogylcans, and angiogenesis, have restored and redensified the tissue volume.^8^ Hylauronic acid is used to enhance volume but has little to no regenerative properties.^9^ The present case report describes a technique in which HA is blended with CaHA–CMC to augment the labia majora in patients following labiaplasty over a 2-month period.

## CASE PRESENTATION

In these 2 cases, 39- and 42-year-old Canadian Caucasian women sought treatment for pronounced labia majora laxity and wrinkling following a labiaplasty procedure performed 6 months prior. A single session of CaHA–CMC and HA was administered using 3 mL of the hybrid filler injected per side; however, the maximum recommended volume to inject is 5 mL per side. Assessment of volume to be injected was determined based on the laxity of the skin, the anatomy (width), and patient desire. To prepare the blended CaHA–CMC and HA filler, a syringe containing 1.5 mL of CaHA–CMC (Radiesse, Merz Aesthetics, Raleigh, NC), 1.0 mL of hyaluronic acid (Belotero Volume, 26 mg/ml, Merz Pharmaceuticals GmbH, Frankfurt, Germany), and 0.5 mL of 1% lidocaine was transferred to a 10 mL syringe using a female-to-female Luer lock adaptor. An empty 10 mL syringe was then connected, and the mixture was exchanged between the syringes at least 10 times to ensure thorough and homogeneous mixing, decreasing the risk of focal accumulations.

Prior to treatment, the labia majora was cleansed with chlorhexidine aqueous solution and the patients were positioned in either lithotomy or frog-leg posture for optimal access. A single-entry point per side was created at the most superior aspect of the labia majora followed by the administration of 0.1 mL of 1% lidocaine with epinephrine for local anesthesia at the anterior entry points. A 23-gauge cannula was then used to deliver the blended mixture via a linear retrograde injection pattern from posterior to anterior in the subdermal plane, depositing thin threads of product for uniform distribution and volumization (Video).

Following injection, patients were advised to apply ice as needed for discomfort. To minimize the risk of complications, strenuous physical activity was restricted for 72 hours and sexual activity avoided for 1 week. Additionally, patients were instructed to refrain from massages and submerging the treatment area in water, including baths, pools, and spas, for 3 days.

Expected postprocedural effects included transient swelling, pain, inflammation, and mild asymmetry, all of which are self-limiting and typically resolve within 2 weeks. Mild swelling and inflammation presented in the 2 patients immediately following injections and were resolved within 2 to 4 days. Patients were counseled on these potential reactions and instructed to follow up if symptoms persisted or worsened beyond the anticipated downtime. Notably, both patients did not report any severe adverse events, such as nodules or vascular complications, at any point throughout the study.

**Figure 1.**
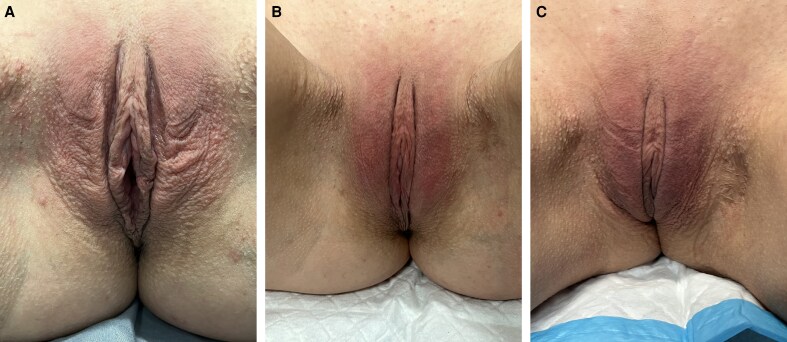
A 39-year-old Caucasian female patient at (A) baseline, (B) 3 months, and (C) 20 months after treatment using a hybrid filler composed of calcium hydroxylapatite–carboxymethycellulose blended with hyaluronic acid.

**Figure 2.**
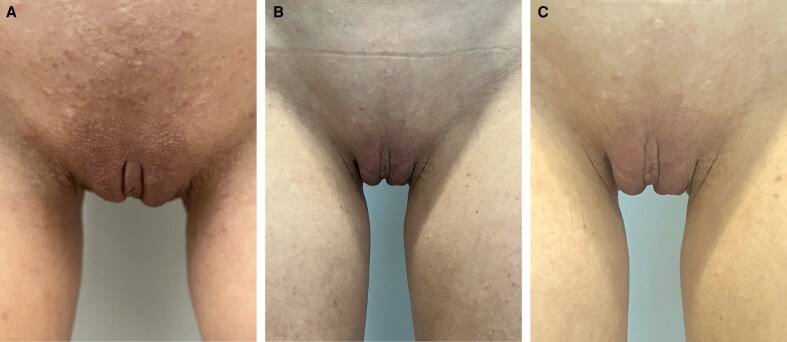
A 42-year-old Caucasian female patient at (A) baseline, (B) 3 months, and (C) 20 months after treatment using a hybrid filled composed of calcium hydroxylapatite–carboxymethycellulose blended with hyaluronic acid.

At the 3- and 20-month follow-ups, both patients demonstrated improvements in skin texture and proportions, with enhanced contour and volume of the labia majora, as assessed by the patient and treatment provider (Figures 1, 2). Overall, patients reported a high level of satisfaction with the aesthetic outcome determined through a direct questioning approach.

## DISCUSSION

Labiaplasty can often result in a decrease in tissue volume and emphasize preexisting laxity of the labia majora. To date, several minimally invasive solutions have been adopted to address these patient concerns focusing individually on the volumizing effects of HA or the regenerative capacity of CaHA–CMC. A promising treatment includes the combination of CaHA–CMC with HA to offer a balanced approach to tissue augmentation, utilizing the strengths of both materials while minimizing their individual limitations, for example, the reduction in volume sometimes reported following the use of CaHA–CMC alone prior to the restoration with newly synthesized collagen. These results would also vary depending on the age of patient, as postmenopausal related reductions in neocollagenesis may impact the predictability of outcome since the effects are dependent on the individual's fibroblast activity and reaction to microspheres.^5^ A recent study compared CaHA–CMC to HA for the treatment of atrophic labia majora and found significant improvements in volumization and flaccidity with both products, however with caveats related to each product depending on case specific presentation. The authors discussed how HA may be suggested for less atrophic patients desiring immediate volumization, whereas CaHA–CMC can be recommended for more advanced atrophy requiring durable dermal remodeling.^10^ The aesthetic and physiological benefits presented here corroborate a previous narrative review in nonlabiaplasty patients that assessed case studies applying CaHA–CMC and HA (22.5 mg/mL) for vaginal rejuvenation and documented improvements on the genital appearance satisfactory score, providing valuable insights for post-labiaplasty augmentation over a longer time-period.^11^

It has been well characterized in literature that HA provides high predictability in results, ensuring consistent volumization,^12^ while CaHA–CMC contributes to longer-lasting effects by stimulating key extracellular matrix components required for enhanced dermal thickness, texture and pliability.^13-15^ By blending the filler and biostimulator together, effectively altering the rheological properties of CaHA–CMC, the CaHA–CMC significantly reduces its elastic modulus (G′) and allows for a more even spread and dispersion of microspheres within the target tissue. The distribution and interspacing of microspheres play a direct role in dermal fibroblast mechanotransduction and activation, with maximal regenerative effects related to neocollagenesis and elastogenesis, and subtle volumization existing in diluted CaHA–CMC with a reduced G′.^16^ The biological half-life of collagen and elastin are primary contributors to the long-term effects following the resorption of both the CMC gel carrier and degradation of HA.

The safety profile and recovery time associated with CaHA–CMC and HA are similarly tolerable, predictable, and more rapid than surgical intervention. However, with any dermal filler, there are inherent risks of short- and long-term complications. Given the abundance of injections in aesthetics, there have been significant effort to identify complication management protocols to mitigate long-lasting effects. For example, bruising is commonly treated by using a cold compress and vitamin K cream. The cold compress and topical vitamin K can similarly be used for postprocedural edema. Moreover, noninflammatory nodule treatment with CaHA can be treated using a 3-step algorithmic approach to resuspend and redistribute the microspheres causing the focal accumulation following mechanical vibration, whereas hyaluronidase is used to resolve nodules caused by HAs.^17,18^ Vascular occlusions are responded to in a similar manner for CaHA–CMC and HA in which hyaluronidase, aspirin, tadalafil, prednisone, hyperbaric chambers, and pentoxifylline can be administered to alleviate symptoms and restore perfusion.^19^ It is important that both the injector and patient receiving the treatment are fully aware of the risks and of the management protocols used to address complications in the circumstance that they arise.

Surgical solutions to labia majora augmentation typically involve a combination of liposuction and autologous or dermal-fat grafting to the area of interest. Despite being beneficial and long-lasting in certain cases, this procedure increases the risk of fat necrosis, increases the required downtime and has higher variability in survival rates due to volume resorption.^20^ Physical restrictions (ie, no strenuous activity or sexual intercourse) are prohibited for an average of 4-8 weeks, with recovery depending on the amelioration of symptoms at both the donor site and labia. Unlike fat transfer, the combination of CaHA–CMC and HA can allow for more cost-effective, controlled, and reliable treatment outcomes. Additionally, the reversibility of HAs offers flexibility, making it possible to adjust or dissolve the filler if needed, whereas fat grafting results are permanent and may fluctuate with weight.

When contrasted to noninvasive procedures such as energy-based devices (eg, radiofrequency) or lasers (eg, CO_2_), the hybrid filler offers more patient comfort during the procedure due to the heat associated with noninvasive treatments, inherently increasing the risk of burns, thermal injury, and dyspigmentation.^21,22^ Moreover, the evidence base for these treatment modalities indicates that the primary therapeutic target is tissue tightening, with no demonstrated impact on augmenting labial volume.^23^ For these reasons, along with the surgical nuances involved, the patients in this report elected to undergo the combination treatment presented here.

Overall, labia majora hypotrophy can contribute to chronic irritation, dryness, discomfort, and pain during intercourse, along with aesthetic concerns that can negatively affect a woman's self-esteem, libido, and overall sexual well-being.^10,24^ Following labia minora reduction, the volumizing and augmenting effects of the hybrid filler treatment for the labia majora presented in this report led to satisfactory patient reported outcomes and improvements to aesthetic appearance, suggesting a potential modality that can be used to mitigate hypotrophic impacts. Further research should explore whether the combined use of CaHA–CMC and HA fillers leads to meaningful improvements in sexual function, confidence, and quality of life.

Notably, several limitations exist for the interpretation of data presented here despite the clinical relevance. This case report included a limited cohort size of patients undergoing labia majora augmentation following labiaplasty that did not represent the full range of Fitzpatrick scale skin types. Increasing and expanding the participants, including comparative groups that did not receive intervention and received only CaHA–CMC or HA, and adopting further quantitative measures of outcomes would strengthen the analyses of the case report, while enabling the ability to tease out specific effects attributed to each product. Moreover, the use of an ultrasound in future studies would further the impact, safety and methodology of this procedure.

## Conclusions

This case report presents outcomes for 2 patients following treatment with a CaHA–CMC and HA hybrid filler for the rejuvenation of the labia majora post-labiaplasty. The procedure was well tolerated with no adverse events reported. This technique may provide a valuable complement to labiaplasty for patients seeking improved aesthetics of the labia majora and vulva without additional surgical intervention. Further complementary studies are required to quantify and validate our findings.
